# Supragingival microbiota, cytokines, and proteins in individuals with different trajectories in experimental gingivitis

**DOI:** 10.1080/20002297.2024.2372861

**Published:** 2024-07-05

**Authors:** Christine Lundtorp-Olsen, Nikoline Nygaard, Laura Massarenti, Florentin Constancias, Christian Damgaard, Ulvi Kahraman Gursoy, Annina van Splunter, Floris J. Bikker, Mervi Gursoy, Merete Markvart, Daniel Belstrøm

**Affiliations:** a Department of Odontology, Section for Clinical Oral Microbiology, Faculty of Health and Medical Sciences, University of Copenhagen, Copenhagen, Denmark; b Department of Odontology, Section for Oral Biology and Immunopathology, Faculty of Health and Medical Sciences, University of Copenhagen, Copenhagen, Denmark; c Institute for Inflammation Research, Center for Rheumatology and Spine Diseases, Rigshospitalet, Copenhagen, Denmark; d Department of Periodontology, Institute of Dentistry, University of Turku, Turku, Finland; e Department of Oral Biochemistry, Academic Center for Dentistry Amsterdam, University of Amsterdam and Vrije Universiteit, Amsterdam, Netherlands

**Keywords:** Gingivitis, periodontitis, microbiota, perturbation, resilience

## Abstract

**Background:**

Gingivitis in response to biofilm formation may exhibit different trajectories. The purposes of the present study were to characterize the composition of the supragingival microbiota and salivary cytokine and protein levels in healthy individuals with different gingivitis patterns, to test the hypothesis that manifestations of gingivitis associate with specific profiles in terms of supragingival microbiota, salivary cytokines, and proteins.

**Methods:**

Forty orally and systemically healthy individuals refrained from all oral hygiene procedures for a period of 14 days, followed by a resolution period of 14 days with regular oral care. Supragingival plaque level and bleeding on probing (BOP) were recorded, and supragingival plaque as well as saliva samples were collected at baseline, day 14, and day 28. Based on change in BOP% from baseline to day 14, rapid (*n* = 15), moderate (*n* = 10), and slow (*n* = 15) responders were identified. Supragingival microbiota composition, salivary cytokine, and protein levels were compared between groups at baseline, day 14, and day 28.

**Results:**

A significantly higher baseline abundance of *Capnocytophaga, Eikenella*, and *Campylobacter* species were recorded in rapid responders, whereas a significantly higher baseline abundance of *Streptococcus* species were detected in slow responders. Slow responders expressed a high degree of resilience, with minimal difference in microbial composition at baseline and after 14 days of resolution (day 28). On the contrary, significant differences in relative abundance of members of the core microbiota, *Streptococcus*, *Actinomyces*, and *Rothia* species, was noted in baseline samples versus day 28 samples in rapid responders. Comparable baseline cytokine and protein levels were recorded in all groups.

**Conclusion:**

Supragingival microbiota composition, but not saliva cytokine and protein profiles, seems to influence the extent of the inflammatory response during development of gingivitis in systemically healthy individuals.

## Key messages

Baseline composition of the supragingival microbiota might predict different gingivitis trajectories.

Microbial resilience after gingivitis might augment oral homeostasis in individuals with a slow gingivitis trajectory.

## Introduction

Gingivitis is the most prevalent oral condition, affecting a substantial part of the population worldwide [1]. If left untreated, gingivitis may progress to periodontitis in susceptible individuals, in particular [2,3].

Gingivitis is perceived as the naturally occurring initial host response towards formation of supragingival biofilm [4], as shown by Harald Löe and co-workers using an experimental gingivitis model [5]. Microbiologically, early microscopy studies showed that gingivitis in response to oral hygiene discontinuation infers not only the amount of biofilm but also induces a shift in the composition of the supragingival biofilm from a situation with scarce amounts of Gram-positive bacteria towards a complex microbial community characterized by colonization of both Gram-positive and Gram-negative bacteria [6].

Recently, contemporary molecular methods have shown that gingivitis is associated with *Fusobacterium* and *Campylobacter* species compared to periodontal health [7,8]. Along this line, longitudinal data reveal that the transition from periodontal health to gingivitis is accompanied by increased abundance of *Actinomyces, Leptotrichia*, and *Fusobacterium* species [9–12].

For decades, gingivitis was considered an unspecific inflammatory reaction, with a universal clinical manifestation, being explained by the unspecific plaque hypothesis [13,14]. However, recent studies have shown clinically different trajectories of gingivitis, where some individuals develop severe inflammation, whereas others only develop modest inflammation in response to biofilm formation on tooth surfaces [15,16]. The biology of these different clinical response patterns is largely uncovered, and it is unknown, if compositional characteristics of the supragingival microbiota and salivary cytokine and protein levels could be either a predisposing or a protective determinant of the periodontal inflammation response.

The purpose of the present study was, therefore, to characterize the composition of the supragingival microbiota and salivary cytokine and protein levels in healthy individuals, who expressed different inflammatory reaction patterns towards a temporary oral hygiene stop. We tested the hypothesis that different trajectories of gingivitis associate with supragingival microbiota profiles and salivary cytokine and protein levels.

## Methods

### Study population

In the present study, data of the placebo group from a double-blinded randomized clinical trial were employed [12]. In brief, the study group was comprised of 40 orally and systemically healthy individuals, recruited at the Department of Odontology, University of Copenhagen in the period from January to May 2022. Inclusion criteria were age 18–35 years and absence of treatment-requiring gingivitis, periodontitis, dental caries, and systemic disease. Exclusion criteria were pregnancy, use of any type of medication, and use of systemic antibiotics in the past 3 months or during the trial.

### Study design

The study design has been described in detail [12]. Briefly, participants refrained from all oral hygiene procedures for a period of 14 days (perturbation period), followed by a resolution period of 14 days, where regular oral hygiene procedures were resumed. The study was approved by the regional ethical committee of the Capital Region of Denmark (H-21003295), reported to the local data authority of University of Copenhagen (514–0434/19–3000), and registered in Clinical Trials.gov (UCPH_01_006). All participants signed informed consent forms, and the study followed the guidelines of the Helsinki Declaration. As the participants were the placebo group in a double-blinded randomized clinical trial, they ingested placebo lozenges twice a day during the trial period of 28 days. The contents of the placebo lozenges were D-mannitol, xylitol, polyvinylpolypyrrolidone, maize starch, citric acid, magnesium salts of fatty acids, lemon flavour, steviol glycoside, and calcium hydrogen phosphate.

### Clinical examination and sample collection

Clinical examination and sample collection have been described [12]. In brief, all participants underwent a complete oral examination prior to participation, confirming the absence of treatment-requiring oral diseases. Next, supragingival biofilm level and bleeding on probing (BOP) were recorded at baseline, day 14, and day 28. Supragingival biofilm was recorded at six sites per tooth using disclosing tablets (SUNSTAR G_U_M®MD RED-COTE®MD), and the amount of supragingival biofilm was scored from 0 to 5 using the modified Quigley-Hein index [17]. BOP was recorded at six sites per tooth and quantified from 0 to 2. In addition, percentages of supragingival biofilm and BOP were calculated. Before using disclosing tablets and before any clinical examination, a pooled supragingival plaque sample and a stimulated saliva sample were collected according to standardized protocols [12]. In brief, supragingival plaque was removed by a sterile curette from the buccal surface of the 2nd quadrant, and saliva was collected using paraffin-mediated chewing stimulation. Supragingival plaque was vortexed in 1 mL saline, and all samples were immediately stored at −18°C and subsequently transferred to −80°C until further analysis.

### Microbial analysis

The laboratory procedures have been described in detail [12]. Briefly, the Illumina MiSeq (Illumina, San Diego, CA, USA) technology was used to sequence the V1–V3 region of the 16S gene with all samples >10.000 reads passing quality control being processed further in downstream analysis. Generated sequences were taxonomically annotated to reference sequences in the extended Human Oral Microbiome RefSeq Database (eHOMD) v 15.2 [18] for identification at highest taxonomic level [12].

### Cytokine analysis

Salivary interleukin (IL)-1β, IL-8, monocyte chemoattractant protein-1 (MCP-1), and macrophage migration inhibitory factor (MIF) levels were measured as previously described [12]. Briefly, after centrifugation of saliva samples at 9300 × *g* for 10 min, cytokines were detected using a bead-based immunoassay. Detection limits were 0.24 pg/mL (IL-1β), 0.36 pg/mL (IL-8), 2.45 pg/mL (MIF), and 0.44 pg/mL (MCP-1). Samples with recordings below detection limit were replaced with limit of detection/2.

### Protein and enzyme analysis

Analysis of proteins and enzymes was performed following manufacturer’s specifications as previously described [12]. First, after centrifugation of saliva at 10,000 × *g* for 10 min, the supernatant was divided into three aliquots, which were stored at − 20°C until further analysis. Total protein concentration was measured using Pierce™ BCA Protein Assay Kit (Thermo Fisher, West Palm Beach, FL, USA, Cat#23227) with Bovine Serum Albumin (BSA) standard from 25 μg/mL to 1500 μg/mL. Amylase activity was tested employing an alpha-amylase substrate consisting of 2-chloro-4-nitrophenyl alpha-D-maltotrioside (Apollo Scientific, Denton, UK, BITJ00020), with absorbance being measured at 450 nm for 15 min with 1 min intervals on a plate reader. Total protease activity (TPA) was recorded using PEK-54 substrate ([FITC]-NIeKKKKVLPIQLNAATDK-[KDbc]) in a working solution of 32 μM diluted in TBS, with proteolytic activity being expressed as the increase in fluorescence per min (F/min). Chitinase activity was recorded using a fluorescence microplate reader with a 360 nm excitation filter and 450 nm emission filter, and fluorescence was recorded for approximately 1 h at 37°C using 5 min intervals. Albumin was measured using microplates coated with rabbit anti-human albumin, cat# A0001 DAKO. Anti-human albumin (horseradish peroxidase) (Biorbyt via Bioconnect, Huissen, The Netherlands, Cat# ORB243267) was used as conjugate, and albumin was detected with o-phenylenediamine dihydrochloride substrate tablets (Thermo Fisher, West Palm Beach, FL, USA, #34006).

### Statistical analysis

Participants were divided into three groups: rapid (*n* = 15), moderate (*n* = 10), and slow responders (*n* = 15), based on their percentage change in BOP% from baseline to week 2.

Multiple linear regression (ANCOVA) with Tukey’s correction was used for analysis of clinical parameters and supragingival biofilm. Kruskal–Wallis tests were used to analyse differences in BOP% between groups at baseline, day 14, and day 28, and a pairwise Wilcoxon test was used to examine differences between groups by day 14.

Normalized sequence data at amplicon sequence variant (ASV) level were used to compute relative abundance, principal component analysis (PCA), Permutational analysis of variance (PERMANOVA), and linear discriminant analysis Effect Size (LEfSe) analysis [18], which were employed for compositional characterization of the supragingival microbiota. Analysis of clinical and microbial data was conducted using a 95% significance level and was run in R v.4.3.1 with RStudio (IDE version 2023.09.1 + 494).

Levels of cytokines (pg/ml), proteases activity (U/ml), and albumin (µg/ml) were compared between groups at each of the three timepoints by means of one-way ANOVA, with Tukey’s correction for multiple comparisons. For the same analytes, comparisons within groups across the three timepoints were carried out with repeated measures one-way ANOVA with Tukey’s correction for multiple comparisons. A *p* value < 0.05 was considered significant for all analyses.

## Results

### Background data

Background data of rapid, moderate, and slow responders are detailed in Table 1. No significant differences in age and sex composition were observed in the groups.

**Table 1.** Background characteristics.

	Slow (*n* = 15)	Moderate (*n* = 10)	Rapid (*n* = 15)
**Age (mean ± SD)**	24.5 (2.3)	24.0 (3.7)	24.3 (3.3)
**Female/male**	9/6	6/4	11/4

### Clinical characteristics

**Figure 1.**
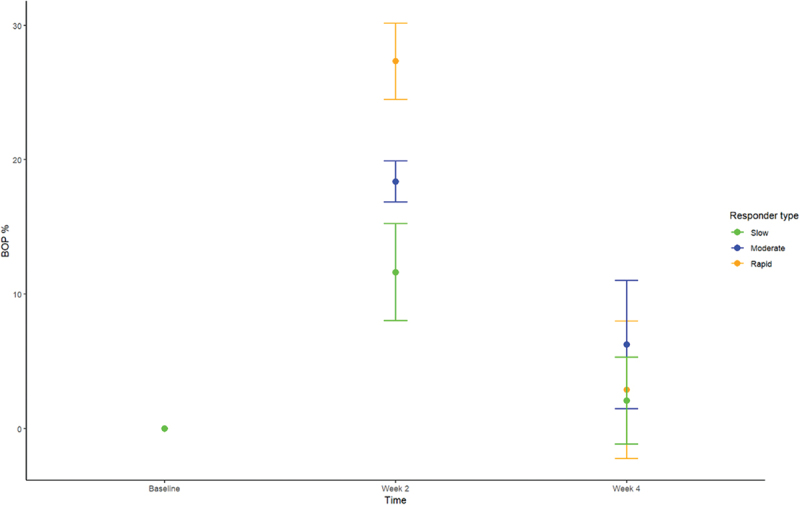
Microbial resolution trajectories after experimental gingivitis. LEfSe analysis of baseline microbiota versus microbiota after 14 days of resolution. a: Slow responders genus level, b: slow responders species level, c: moderate responders genus level, d: moderate responders species level, e: rapid responders genus level, f: rapid responders species level (baseline: brown, week 4: grey).

Data on supragingival biofilm level and BOP% are presented in Tables 2 and 3, respectively. No significant difference in supragingival biofilm score was observed in slow, moderate, and rapid responders at baseline, day 14, and day 28. No significant difference in BOP% was recorded at baseline and day 28. Relative change in BOP% in rapid, moderate, and slow responders is visualized in Figure 1.

**Table 2.** Supragingival biofilm expressed and mean ± SD at baseline, week 2, and week 4.

	Slow	Moderate	Rapid
Baseline	0.9 (0.3)	0.9 (0.4)	1 (0.4)
Week 2	2.5 (0.4)	2.5 (0.4)	2.6 (0.3)
Week 4	0.8 (0.3)	1.2 (0.5)	1 (0.3)

**Table 3.** Percentage of sites with bleeding on probing (BOP%) expressed and mean ± SD at baseline, week 2, and week 4.

	Slow	Moderate	Rapid
Baseline	5.0 (4.5)	5 (3.1)	5.9 (4)
Week 2	16.6 (5.4)	23.4 (3.4)	33.2 (4.7)
Week 4	7.1 (4.9)	11.3 (5.5)	8.8 (4.8)

### Baseline composition of the supragingival microbiota

Baseline relative abundance of predominant bacterial genera and species is presented in Figure 2a-b. Significantly higher abundances of genus *Capnocytophaga* were observed in samples from rapid responders (11.2%), as compared to moderate (7.3%) and slow responders (5.5%). In slow responders, a significantly higher abundance of genus *Streptococcus* (11.7%) was recorded, compared to moderate (7.3%) and rapid responders (7.0%). PCA showed no separate clustering (Figure 2(c)), and PERMANOVA detected no significant compositional difference between groups. LEfSe analysis showed that genus *Streptococcus* was associated with slow responders, whereas genus *Veillonella* was associated with moderate responders, and genera *Capnocytophaga* and *Eikenella* were associated with rapid responders (Figure 2(d)). At species level, *Eikenella corrodens*, *Gemella morbillorum,* and *Campylobacter showae* were associated with rapid responders, whereas different ASVs of *Veillonella parvula* were associated with moderate and slow responders (Figure 2(e)).

**Figure 2.**
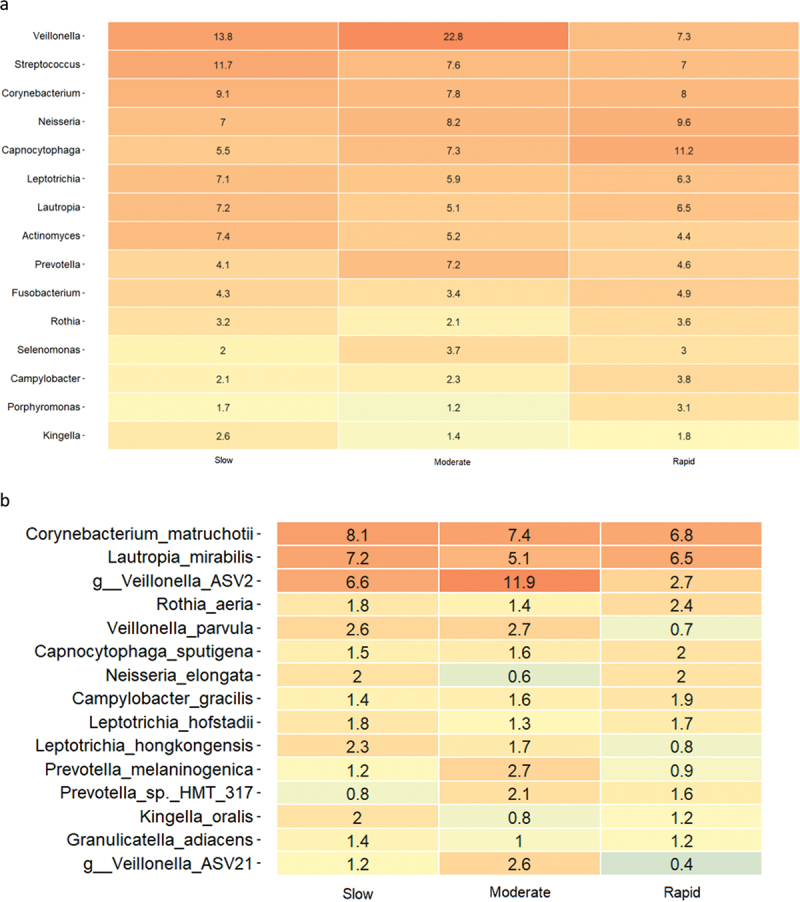
Baseline composition of the supragingival microbiota. a: Mean levels of predominant 15 genera, b: mean levels of 15 predominant species, c: principal component analysis, d: LEfSe genus level, e: LEfSe species level (slow: green, moderate: blue, rapid: yellow).

**Figure 2.**
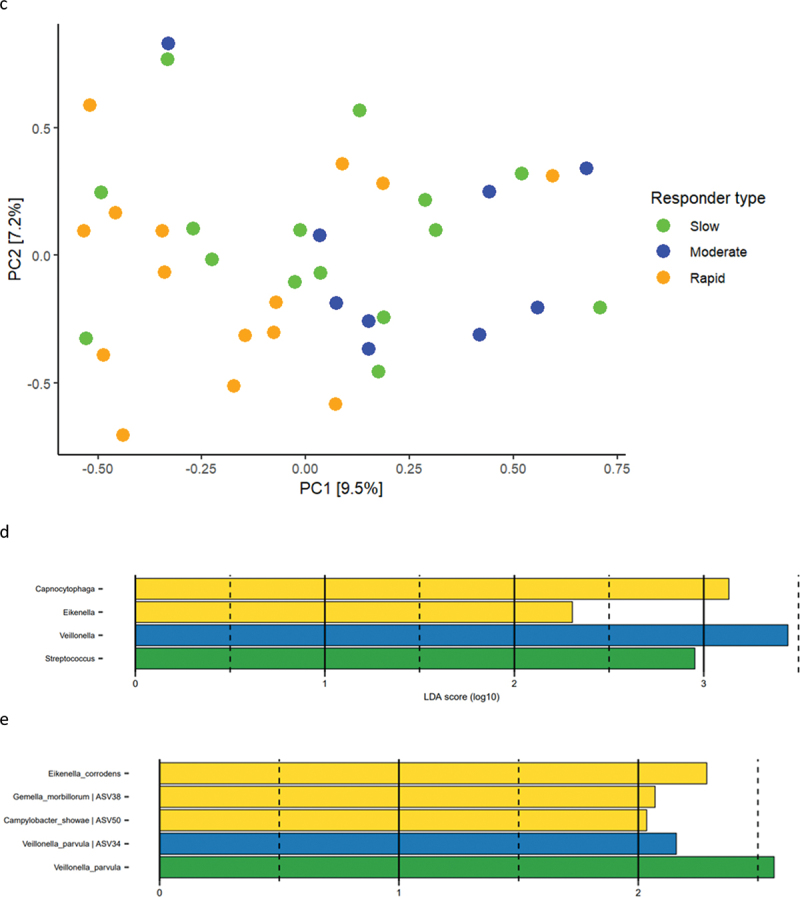
(Continued).

### Supragingival microbiota in experimental gingivitis

Relative abundance of predominant bacterial genera and species after 14 days of oral hygiene discontinuation is presented in Figure 3a-b. Significantly higher abundance of genus *Capnocytophaga* was observed in samples from slow (15.9%) and moderate responders (15.5%) versus rapid responders (11.6%). PCA showed no clustering of samples (Figure 3(c)), and PERMANOVA revealed no significant differences between groups. LEfSe analysis showed no differences at either genus or species level.

**Figure 3.**
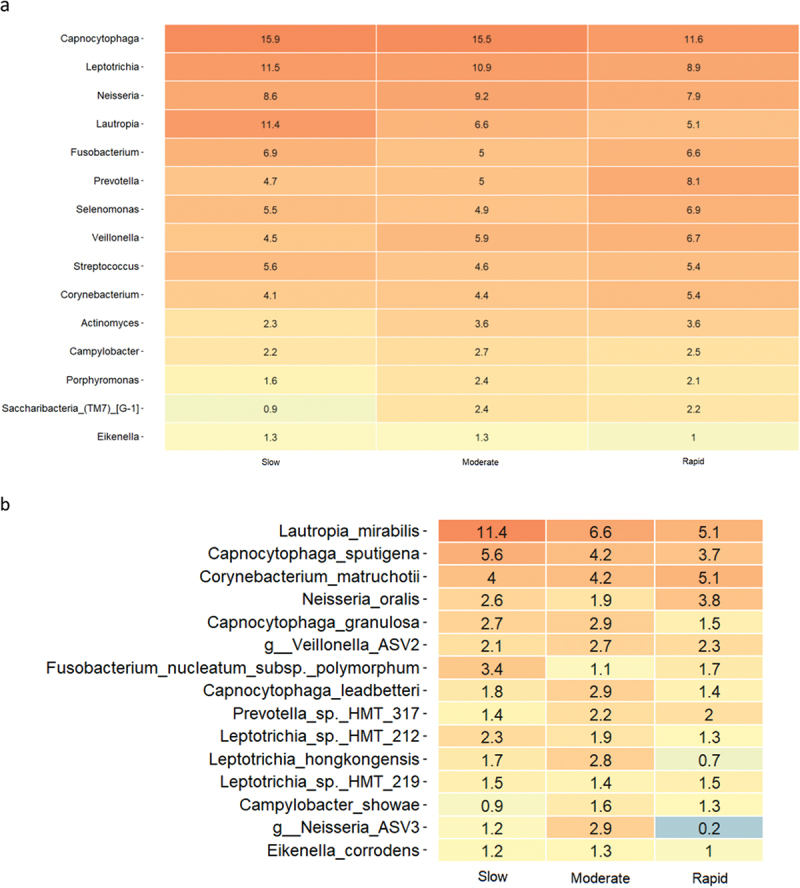
Supragingival microbiota in experimental gingivitis. a: Mean levels of predominant 15 genera, b: mean levels of 15 predominant species, c: principal component analysis (slow: green, moderate: blue, rapid: yellow).

**Figure 3.**
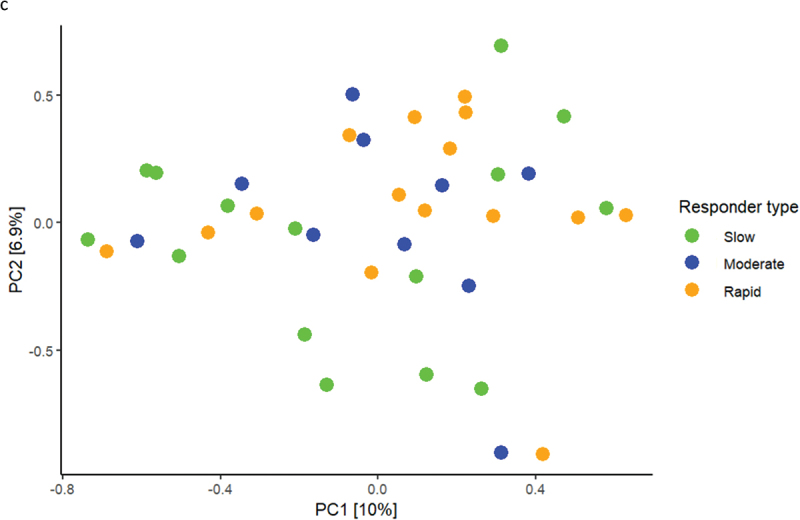
(Continued).

### Supragingival microbiota after resolution of experimental gingivitis

Relative abundance of predominant bacterial genera and species after 14 days of resolution is presented in Figure 4a-b. PCA revealed no clustering of samples (Figure 4(c)), and PERMANOVA detected no significant difference in microbial composition between groups. LEfSe analysis showed that genus *Eikenella* and genus *Pseudopropionibacterium* were associated with rapid responders (Figure 4(d)). At species level, *Saccharibacteria_TM7_G1_HMT_347* and *Leptotrichia wadei* were associated with moderate responders, whereas *Eikenella corrodens* and *Corynebacterium durum* were associated with rapid responders (Figure 4(e)).

**Figure 4.**
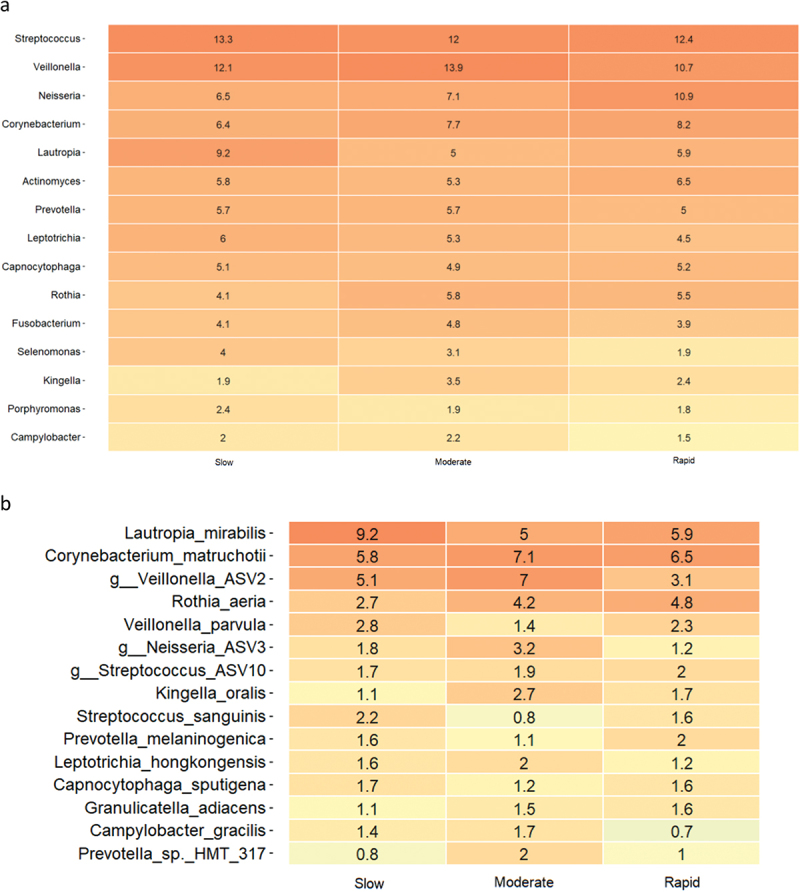
Supragingival microbiota after resolution of experimental gingivitis. a: Mean levels of 15 predominant genera, b: mean levels of 15 predominant species, c: principal component analysis, d: LEfSe genus level, e: LEfSe species level (slow: green, moderate: blue, rapid: yellow).

**Figure 4.**
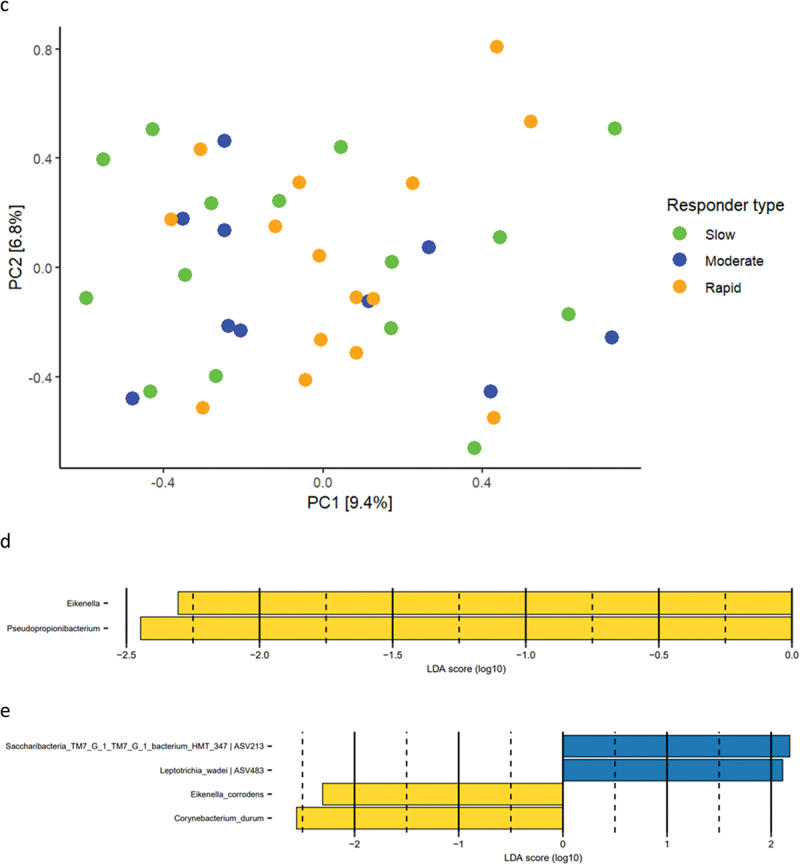
(Continued).

### Microbial resolution trajectories after experimental gingivitis

LEfSe analysis of the supragingival microbiota in baseline conditions versus 14 days of resolution after oral hygiene discontinuation (day 28) revealed minor differences in slow responders as evaluated at genus and species level (Figure 5a-b). In contrast, multiple bacterial genera and species differentiated baseline conditions from day 28, in moderate and rapid responders (Figure 5(c-f)).

**Figure 5.**
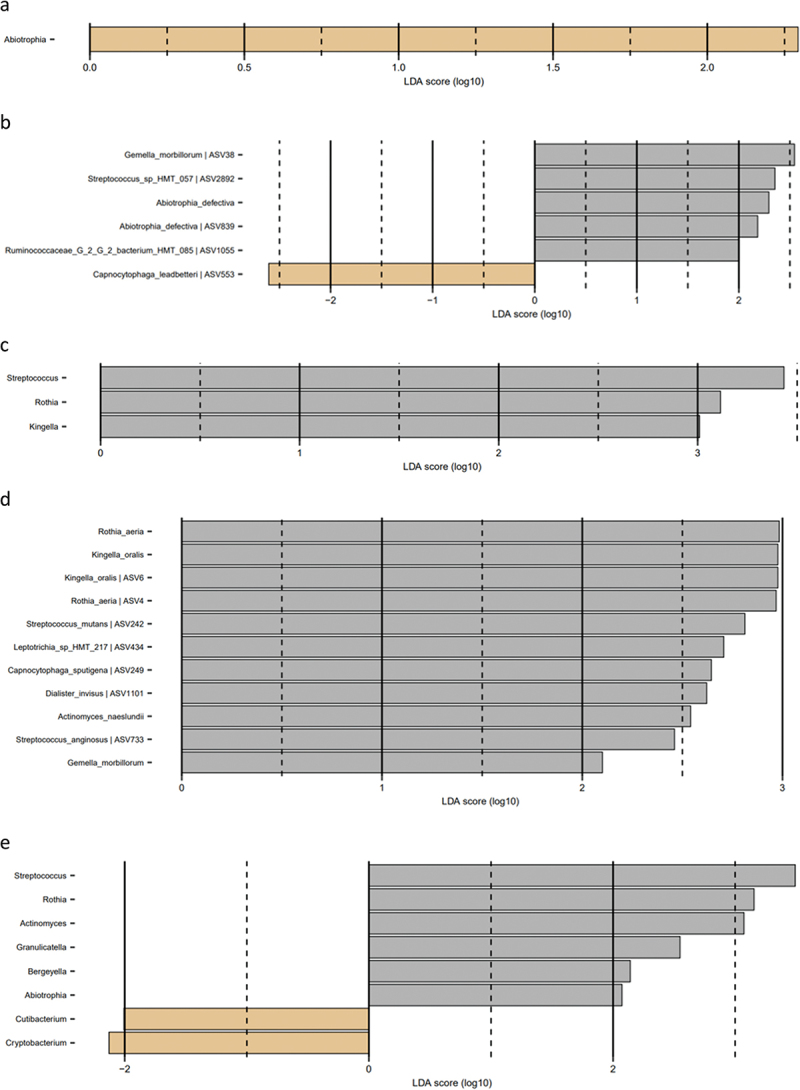
Cytokines, protease activity, and albumin between groups at each timepoint. Levels of a: IL-1β, b: IL-8, c: MIF, d: MCP-1, e: amylase activity, f: chitinase activity, g: total protease activity, and h: albumin levels in slow (green), moderate (blue), and rapid responders (orange) at baseline (T1), day 14 (T2), and day 28 (T3). Bars represent mean values. Comparisons between groups at each timepoint were carried out on log10-transformed values via one-way ANOVA with Tukey’s correction for multiple testing. **p < 0.01.

### Salivary cytokine and protein levels

While slow and moderate responders had similar baseline levels of the measured cytokines, the rapid responders tended to have lower levels than the other group. However, this trend only reached significance for comparison between moderate and rapid responders for IL-8 (*p* = 0.004, Figure 5(b)). No significant differences in cytokine levels between groups were observed at day 14 and day 28.

Levels of amylase, chitinase, and TPA were not significantly different between groups at any of the timepoints (Figure 5(e-g)). The same was observed for albumin levels (Figure 5(h)).

**Figure 6.**
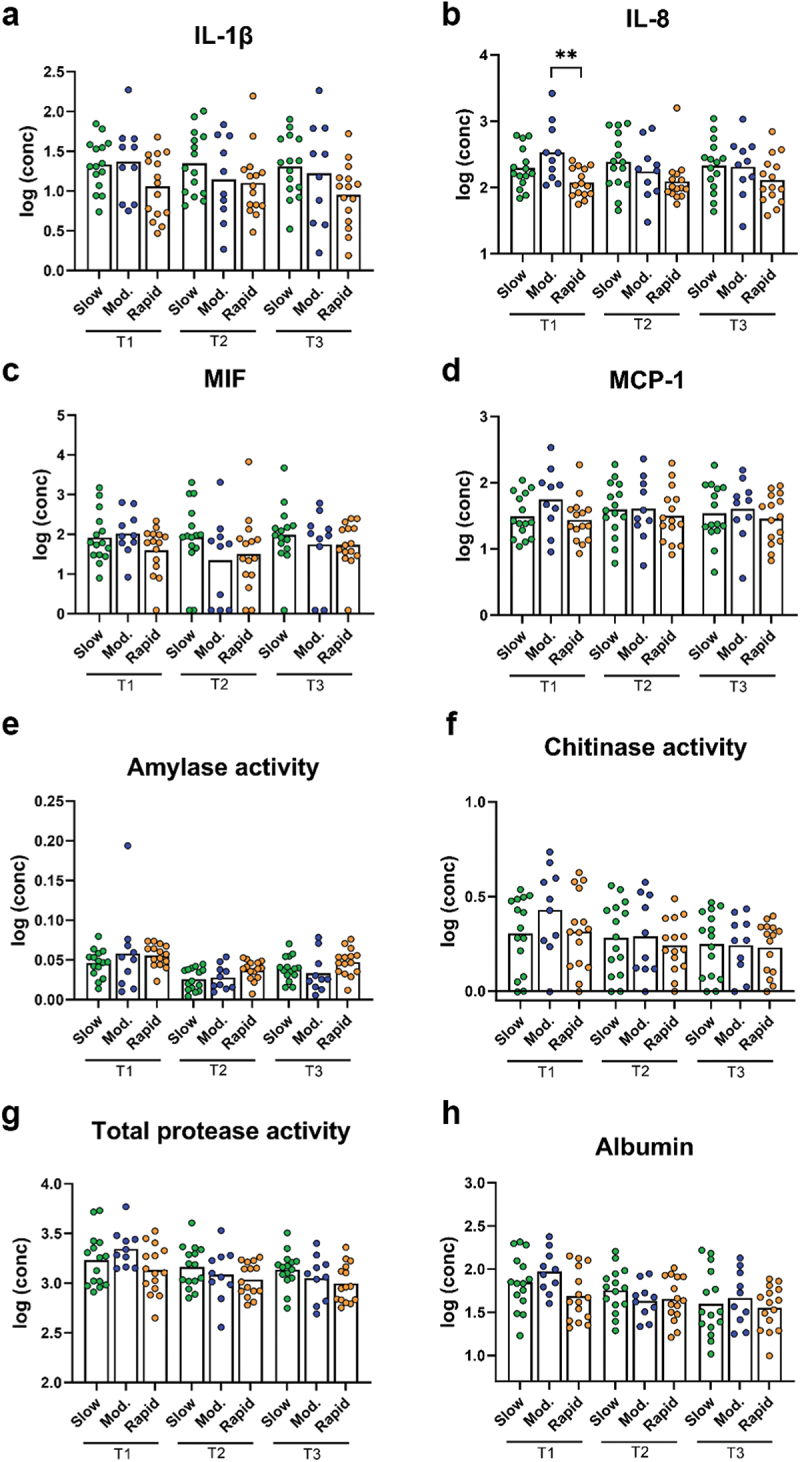
Cytokines, protease activity, and albumin levels within groups at each timepoint. Levels of a: IL-1β, b: IL-8, c: MIF, d: MCP-1, e: amylase activity, f: chitinase activity, g: total protease activity, and h: albumin levels in slow (green), moderate (blue), and rapid responders (orange). Bars represent mean values. Comparisons between timepoint for each group were carried out on log10-transformed values via repeated measures one-way ANOVA with Tukey’s correction for multiple testing. *p < 0.05, **p < 0.01, ***p < 0.001, and ****p < 0.0001.

Cytokine levels were generally stable for slow and rapid responders. On the other hand, moderate responders had significantly lower levels of IL-8 at day 14 compared to baseline (*p* = 0.01, Figure 6(b)), and the same pattern was observed for MIF (*p* = 0.02, Figure 6(c)).

In slow responders, amylase activity was significantly lower at day 14 compared to baseline and to day 28 (*p* < 0.0001 and *p* = 0.003, respectively, Figure 6(e)). The same pattern was observed for rapid responders (*p* < 0.0001 for baseline vs day 14 and *p* = 0.02 for day 14 vs day 28, respectively, Figure 6(e)). Chitinase activity was lower at day 14 and day 28 compared to baseline in moderate responders (*p* = 0.01 and *p* = 0.002, respectively, Figure 6(f)), and the same pattern was observed in the rapid responders (*p* = 0.02 and *p* = 0.008, respectively, Figure 6(f)). TPA followed the same trends as chitinase, but significant differences were only observed between day 28 and baseline for both moderate and rapid responders (*p* = 0.03 and *p* = 0.02, respectively, Figure 6(g)). Albumin levels were decreased at day 28 compared to baseline in slow responders (*p* = 0.009, Figure 6(h)) and in moderate responders (*p* = 0.02, Figure 6(h)). In moderate responders, levels were also decreased at day 14 compared to baseline (*p* = 0.01, Figure 6(h)).

**Figure 5.**
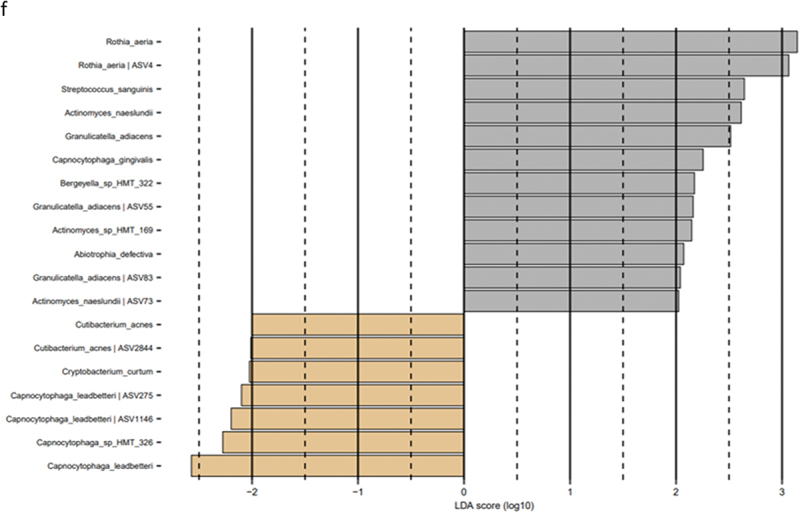
(Continued).

**Figure 7.**
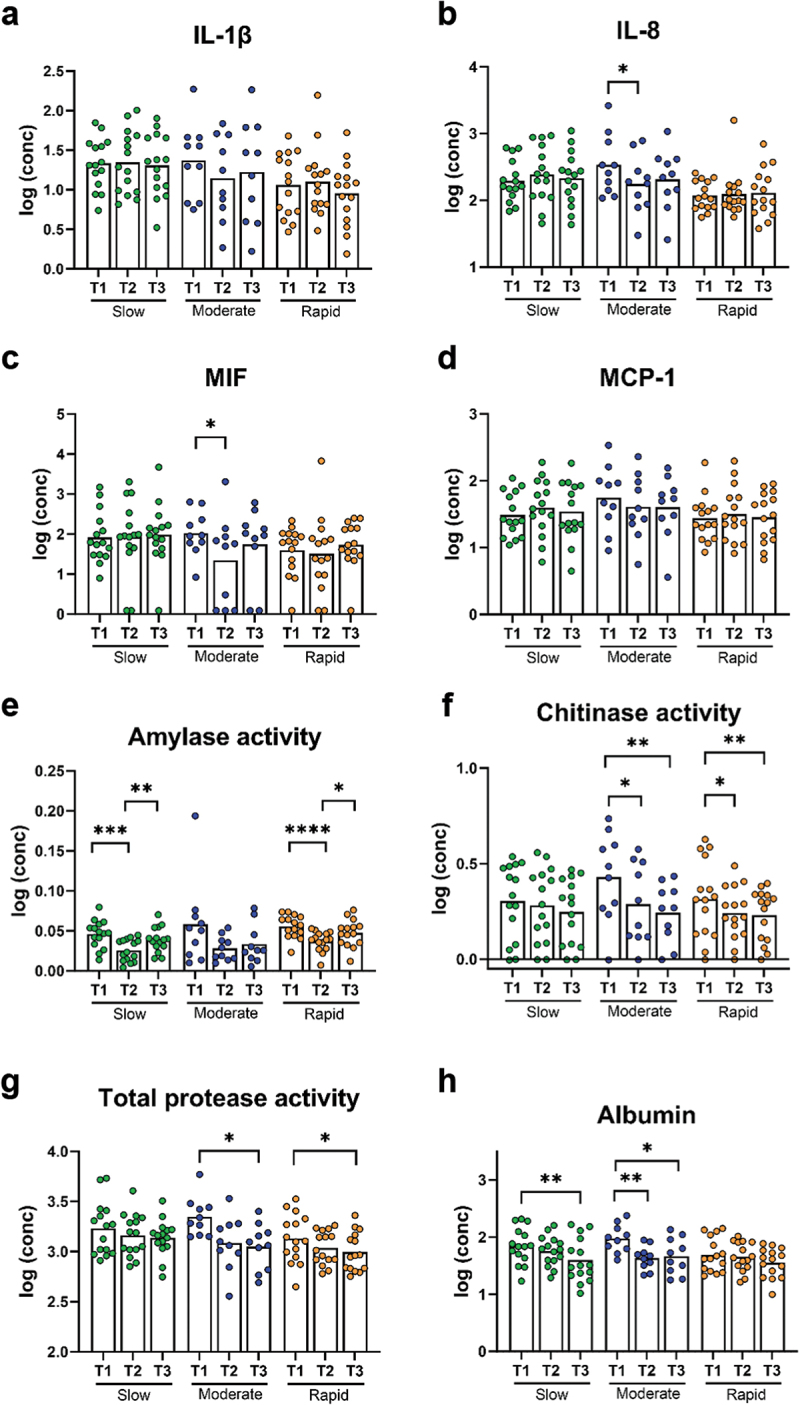
Cytokines, protease activity and albumin levels within groups at each timepoint. Levels of a: IL-1β, b: IL-8, c: MIF, d: MCP-1 (D), e: amylase activity, f: chitinase activity, g: total protease activity, and h: albumin levels in slow (green), moderate (blue) and rapid responders (orange). Bars represent mean values. Comparisons between timepoint for each group were carried out on log10-transformed values via repeated measures One-way ANOVA with Tukey’s correction for multiple testing. *: *p* < 0.05, **: *p* < 0.01. ***: *p* < 0.001 and ****: *p* < 0.0001.

## Discussion

Findings from the present study confirmed the hypothesis, as different trajectories of experimental gingivitis were associated with baseline composition of the supragingival microbiota. On the other hand, salivary cytokine and protein levels did not differentiate slow, moderate, and rapid responders before, during, or after perturbation by experimental gingivitis.

The primary finding was that the main microbial difference was observed in baseline healthy conditions, with *Streptococcus* species being associated with slow responders, whereas *Campylobacter, Eikenella*, and *Capnocytophaga* were associated with rapid responders (Figure 2(c,d)). Notably, *Campylobacter, Eikenella*, and *Capnocytophaga* are Gram-negative facultative anaerobic bacteria, which have been described to associate with periodontitis [19–22]. In contrast, *Streptococcus* species are reported to be less abundant in the periodontal pocket as compared to other oral sites, and genus *Streptococcus* contains several members known to be critically involved in the caries process [23,24]. Hence, our data reveal that oral health may be comprised by different composition characteristics of the supragingival microbiota, with a predominance of bacterial species associated with either periodontal inflammation or dental caries. Along this line, previous studies have reported different salivary metabolome characteristics [25], as well as significant variation in salivary cytokine levels [26] in orally healthy individuals. Hence, our data in concert with other reports reveal that oral health can be composed by different versions of symbiosis [25,26].

Another important finding was that the major difference in clinical inflammation observed in slow (BOP% = 16.6%), moderate (BOP% = 23.4%), and rapid responders (BOP% = 33.2%) after 14 days of oral discontinuation (Table 3, Figure 1) could not be predicted by baseline levels of biofilm and BOP%, which was insignificant in the three groups (Tables 2 and 3). In addition, no difference was observed in supragingival biofilm levels on day 14. Microbiologically, only minor differences were observed at day 14, with data on relative abundance, PCA, PERMANOVA, and LEfSe data all pointing towards a convergence of the composition of the microbiota in individuals with different gingivitis response patterns (Figure 3a-c). In general, these findings are in line with data from a recent study performed in 21 healthy individuals [15]. Therefore, data suggest that compositional changes of the supragingival microbiota during gingivitis follow a preprogrammed pattern, irrespective of baseline microbial composition, which is characterized predominantly by an increase in *Capnocytophaga* species together with a decrease in abundance of *Streptococcus* species.

Interestingly, two recent studies have reported that gingival crevicular fluid (GCF) cytokine levels are associated with different clinical trajectories of gingivitis [15,16]. In our study, we were not able to confirm these findings, as similar cytokine and protein levels were recorded at baseline in all groups (Figure 5). In addition, minimal differences in cytokine and protein levels between groups were observed after 14 days of oral hygiene stop, whereas significant differences were observed within each group at day 14 and day 28, as compared to baseline conditions (Figure 6). As such, we were not able to confirm previous findings from GCF using saliva, which most likely is the consequence of different origins of cytokines in GCF and saliva, as previously suggested [27]. Nevertheless, it seems that despite baseline supragingival microbial composition being a potential contributing factor in different gingivitis trajectories, the individual trajectory is probably the consequence of host modulation, rather than different microbial trajectories from health to gingivitis. Indeed, this is in line with the inflammation-mediated polymicrobial-emergence and dysbiotic-exacerbation (IMPEDE) theory, which denotes the inflammatory response as the determining factor in development of periodontal inflammation and progression from gingivitis to periodontitis [28].

Resilience of the oral microbiota has been named as a key component in the maintenance of oral health [29,30]. In general, the oral microbiota is reported to express short- and long-term stability with personalized characteristics, as long oral health is maintained [31–33]. Along this line, we have previously reported the supragingival microbiota in orally healthy individuals to be highly resilient towards temporary perturbations such as sugar stress and oral hygiene discontinuation [12,34]. It is, therefore, interesting that data from the present study clearly demonstrate minimal differences in baseline microbial composition versus microbial composition after 14 days resolution in the slow responder group (Figure 5a,b), as compared to the moderate responder group (Figure 5c-d), as well as the rapid responder group, where persistent changes in core members of the microbiota such as *Streptococcus*, *Actinomyces*, and *Rothia* species were observed (Figure 5e,f). Hence, our data suggest that slow responders were more capable of returning to baseline microbial conditions than moderate and rapid responders. From a microbiological point of view, slow responders may, therefore, be better protected against future perturbations incited by irregular oral hygiene. However, it remains to be elucidated, if severe gingivitis predisposes to or protects against progression to periodontitis.

The present study has some limitations, including the fact that oral hygiene discontinuation was only maintained for 14 days, as compared to the original protocol of 21 days [5], which means that details on sustained gingivitis might have been lost. However, because the focus of the present study was primarily on the role of the oral microbiota in gingivitis, we deliberately chose a 14-day perturbation. In addition, the study cohort was comprised of young individuals with excellent oral health, which hampers the external validity of the data presented. However, from an ethical point of view, we considered it unjustifiable to induce gingivitis in a more representative but also more disease-prone study cohort. Finally, it should be noted that experimental gingivitis does not reflect the conditions of manifesting periodontal inflammation [35], which means that data from the present study are most likely not be representative of either an established gingivitis lesion nor an initial periodontitis lesion.

In conclusion, data from the present study showed that individuals with a slow trajectory of gingivitis were associated with higher abundance of *Streptococcus* species and lower abundance of *Capnocytophaga, Eikenella*, and *Campylobacter* species compared to moderate and rapid responders. In addition, slow responders demonstrated the highest degree of microbial resilience towards experimental gingivitis incited by oral hygiene discontinuation. On the contrary, salivary cytokine and protein levels did not differentiate slow, moderate, and rapid responders before, during, or after experimental gingivitis. Future studies using more advanced molecular techniques such as metagenomics, metatranscriptomics, and metaproteomics are needed to fully reveal the potential role of bacterial gene expression and the host-mediated response in clinically different trajectories of gingivitis.

## Data Availability

Publicly available data were retrieved from European Nucleotide Archive (ENA) from accession number PRJEB69273.
